# Recovery of α-L-fucosidase in fucosidosis nonsense variants by readthrough stimulation and release factor degradation

**DOI:** 10.1242/dmm.052495

**Published:** 2026-01-07

**Authors:** Hannah Bäumges, Dennis Lebeda, Philip Deppe, Mai-Britt Ilse, Sven Thoms, Torben Lübke

**Affiliations:** ^1^ Biochemistry III, Department of Chemistry, Bielefeld University, 33615 Bielefeld, Germany; ^2^ Biochemistry and Molecular Medicine, Medical School OWL, Bielefeld University, 33615 Bielefeld, Germany

**Keywords:** Fucosidosis, α-L-fucosidase, Translational readthrough, Nonsense mutations, Premature termination codons

## Abstract

Fucosidosis is an ultra-rare and fatal lysosomal storage disease caused by the impaired lysosomal degradation of fucosylated glycoconjugates due to a deficiency in the lysosomal tissue α-L-fucosidase (FUCA1). The accumulation of fucosylated metabolites within lysosomes leads to a range of severe, primarily neurological, symptoms, including cognitive impairment and progressive motor dysfunction. In this study, we explored a therapeutic approach using translational readthrough (TR) for patients with premature termination codons resulting from nonsense mutations in the *FUCA1* gene. We ectopically expressed several clinically identified FUCA1 nonsense variants in a cell line with low endogenous FUCA1 expression. Treatment with the aminoglycoside G418 induced TR, leading to partial recovery of the full-length enzyme and FUCA1 activity. Moreover, combining aminoglycoside treatment with CC-885-induced degradation of the eukaryotic release factor subunit eRF3a further enhanced FUCA1 restoration in two variants (p.Q82X and p.W188X). This study lays the groundwork for individualized TR therapy for patients with fucosidosis with *FUCA1* nonsense variants.

## Teaser

**Summary:** A combination of readthrough stimulation and release factor degradation can restore α-L-fucosidase function in cell reporter models of the lysosomal storage disease fucosidosis.

## INTRODUCTION

The lysosomal enzyme α-L-fucosidase (FUCA1; EC 3.2.1.51; CAZy family GH29) removes fucose residues from fucosylated glycoconjugates, and its deficiency leads to the ultra-rare lysosomal storage disease (LSD) fucosidosis [Online Mendelian Inheritance in Man (OMIM) 230000], which is characterized by the progressive accumulation of fucosylated glycopeptides and glycolipids in urine and in lysosomes of many tissues (Michalski and Klein, 1999; Willems et al., 1991). Fucosidosis patients develop a wide and heterogeneous spectrum of clinical features, including neurodegeneration with mental and motor deterioration. Patients may also exhibit coarse facies, growth retardation, recurrent infections, dysostosis multiplex and angiokeratoma corporis diffusum (Willems et al., 1991, 1999).

The human *FUCA1* gene spans 23 kb on chromosome 1 and is composed of eight exons (NCBI NM_000147.5). After cleavage of the 27-amino-acid signal peptide and N-glycosylation, the mature FUCA1 protein comprises 439 amino acids with a molecular mass of ∼50-55 kDa (NCBI NP_000138.2) and finally assembles into a homotetramer (Armstrong et al., 2022).

Fucosidosis is caused by homozygous or, in rare cases, compound heterozygous mutations in the *FUCA1* gene, which lead to a severely reduced function or loss of function of the FUCA1 protein. To date, at least 37 such pathogenic variants have been reported (Willems et al., 1999; Stenson et al., 2020; Stepien et al., 2020). Among these pathogenic variants, 11 nonsense mutations cause the introduction of premature termination codons (PTCs) (Stenson et al., 2020; Domin et al., 2021; Pekdemir et al., 2025). Depending on their position within the gene, these PTCs can lead to nonsense-mediated mRNA decay (NMD), thus preventing protein synthesis. In cases in which NMD is inefficient or inactive, truncated and non-functional FUCA1 proteins may be produced.

Translational readthrough (TR) describes the decoding of a stop as a sense codon during translational termination by binding of a near-cognate tRNA to a stop codon instead of the termination complex consisting of eukaryotic release factor (eRF1; also known as ETF1) and eRF3a (also known as GSPT1). As a result, the translational elongation continues until the ribosome encounters the next in-frame stop codon (Martins-Dias and Romão, 2021). TR can be induced by treatment with small molecules, also termed TR-inducing drugs (TRIDs), such as the aminoglycosides gentamicin or geneticin (G418), as these increase the likelihood of near-cognate tRNA incorporation at stop codons during translation (Prokhorova et al., 2017).

This mechanism has been tested as a potential therapeutic approach to bypass PTCs and restore the full-length protein in various LSDs, such as Fabry disease and aspartylglucosaminuria, as well as in many non-LSD monogenetic diseases (Lombardi et al., 2020a; Banning et al., 2018; Gómez-Grau et al., 2015). Whether TR can be efficiently induced at a (premature) stop codon depends on the stop codon itself. UGA is the most readthrough-prone (leaky) stop codon, UAG is intermediate, and UAA is the most efficient (tightest) stop codon. Furthermore, TR efficiency is strongly influenced by the surrounding nucleotide context. Particularly, the nucleotide immediately downstream of the stop codon plays a key role, with cytosine (C) at this position often associated with increased TR efficiency. This sequence-dependent influence on TR is known as the stop codon context (SCC) and is typically defined by the stop codon together with the ∼20 adjacent nucleotides (ten on each side of the stop codon). Certain SCCs are more prone to readthrough, making them a critical factor in the development and evaluation of TR assays. In particular, the high-readthrough motif UGA CUAG is associated with high TR inducibility (Lebeda et al., 2024; Schueren et al., 2014; Wangen and Green, 2020; Loughran et al., 2023; Smoljanow et al., 2025).

However, the clinical use of aminoglycosides (and other TRIDs) as patient-specific, long-term treatment remains problematic as they are associated with ototoxicity and nephrotoxicity (Lacy et al., 1998). Considering the potent TR-inducing effects of aminoglycosides, a promising approach would be to reduce the TRID concentration required to achieve a therapeutic level of full-length protein. Recent studies analyzed this strategy by using the small molecule CC-885, which leads to degradation of eRF3a (Baradaran-Heravi et al., 2021; Heldin et al., 2023). Individual therapies based on TR could be specifically suitable for fucosidosis and other LSDs, particularly in cases caused by PTCs. Depending on the SCC, TR occurs at up to tenfold higher rates at PTCs than at natural stop codons and can therefore be induced to levels ≥1% (Lombardi et al., 2020b; Schilff et al., 2021).

In this study, we analyzed pathological nonsense mutations in the *FUCA1* gene to investigate the potential for TR induction and functional protein recovery by aminoglycoside treatment alone or in combination with eRF3a degradation in a cell culture system. We show that G418 restores FUCA1 protein expression and FUCA1 activity, and that its combination with eRF3a degradation amplifies this effect synergistically, highlighting its potential for individualized therapeutic strategies.

## RESULTS

### Aminoglycoside stimulation induces translational readthrough and the recovery of full-length FUCA1

**Fig. 1.**
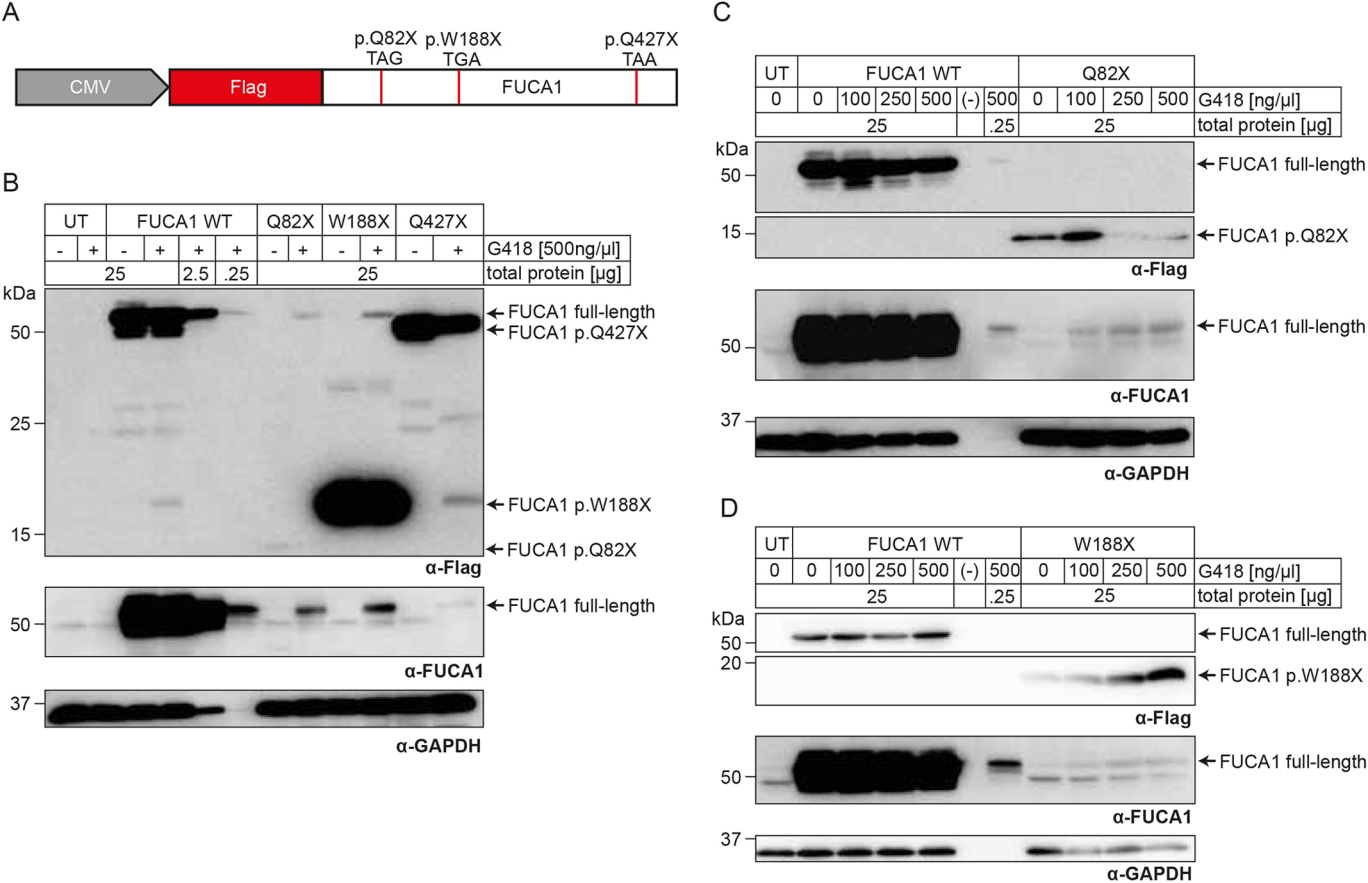
**Induction of translational readthrough leads to recovery of full-length α-L-fucosidase (FUCA1).** (A) Schematic structure of our *FUCA1* constructs containing one of the three analyzed premature termination codons (PTCs) with their stop codon sequence. CMV, cytomegalovirus. (B-D) HT1080 cells were left untransfected (UT) or were transiently transfected with different constructs encoding FUCA1 variants. Cells were treated with various concentrations of geneticin (G418). FUCA1 p.Q82X is expected at 7 kDa, FUCA1 p.W188X at 19 kDa and FUCA1 p.Q427X at 47 kDa. Cleared lysates were obtained and subjected to SDS-PAGE followed by western blot analysis using anti-Flag-tag and anti-FUCA1 antibody. α-GAPDH was used as a loading control. ‘−’ indicates an empty lane. (C,D) Representative western blots from one of three biological replicates.

Of the 11 known pathogenic nonsense mutations of the human *FUCA1* gene (Willems et al., 1999; Stepien et al., 2020), three were further analyzed to evaluate their potential for translational readthrough. We analyzed these three mutations based on their stop codon type and their position within the gene. The three mutations examined were as follows: p.Q82X (c.244C>T), located on exon 1 and introduces a UAG stop codon; p.W188X (c.564G>A), located on exon 3 and results in a UGA stop codon; and p.Q427X (c.1279C>T), located on exon 8 and causes a UAA stop codon (Fig. 1A). None of these three variants would be expected to promote TR, as their surrounding sequences showed no similarity to the known high-readthrough motif UGA CUAG (Martins-Dias and Romão, 2021; Schueren et al., 2014).

To test whether TR can be induced at the fucosidosis stop mutations, we transiently transfected HT1080 cells with plasmids encoding N-terminally Flag-tagged *FUCA1* full-length cDNA, wild type (WT) or one of the three selected PTC mutations (Fig. 1A). HT1080 cells were chosen owing to their low endogenous expression of *FUCA1* and low FUCA1 activity (Fig. 1B-D; Fig. S1). Furthermore, this cell line exhibits robust transfection efficiency and is permissive to aminoglycoside-induced TR, which are prerequisites for PTC suppression strategies. Western blot analyses with antibodies targeting the Flag-tag or FUCA1 confirmed high levels of FUCA1 expression in WT-transfected cells (Fig. 1B). The observed apparent molecular mass of FUCA1 variants was slightly higher than the predicted size owing to post-translational N-glycosylation (∼2-3 kDa). We loaded 25 µg FUCA1-WT cell lysates, together with 2.5 µg (10% load) and 0.25 µg (1% load), to be able to compare them with full-length restored FUCA1 PTC variants after treatment. In untreated cells transfected with any of the PTC variants, only truncated FUCA1 was expressed. Upon treatment with 500 ng/µl G418, full-length FUCA1 was partially restored in all PTC variants using 25 µg total protein. The p.Q427X sample showed the weakest recovery, with a faint band just above 50 kDa when tested with the anti-FUCA1 antibody. Further, a truncated FUCA1 variant appeared upon G418 treatment in wild-type and p.Q427X-transfected cells, indicating a degradative effect of G418 on FUCA1 (Fig. 1B). TR induction recovers observable levels of full-length FUCA1 at the p.Q82X and p.W188X PTCs.

We next wanted to determine the aminoglycoside concentration critical for the stimulation of TR in these constructs. We treated untransfected and transfected cells with various G418 concentrations. Expectedly, untransfected cell lysates revealed very low endogenous FUCA1 expression, and untreated p.Q82X (Fig. 1C) and p.W188X (Fig. 1D) lysates showed truncated but no full-length FUCA1. We found full-length FUCA1 recovery for both constructs at 100 ng/µl G418. More intense FUCA1 signals were observed at higher G418 concentrations (Fig. 1C,D). The detection of full-length FUCA1 recovery at G418 below 500 ng/µl required the anti-FUCA1 antibody, most likely owing to higher affinity to FUCA1 compared to the anti-Flag antibody (Fig. 1C). This interpretation is supported by the signal intensities of FUCA1 WT using anti-Flag and anti-FUCA1 antibody (Fig. 1C).

### The small molecule CC-885 increases the effect of translational readthrough

**Fig. 2.**
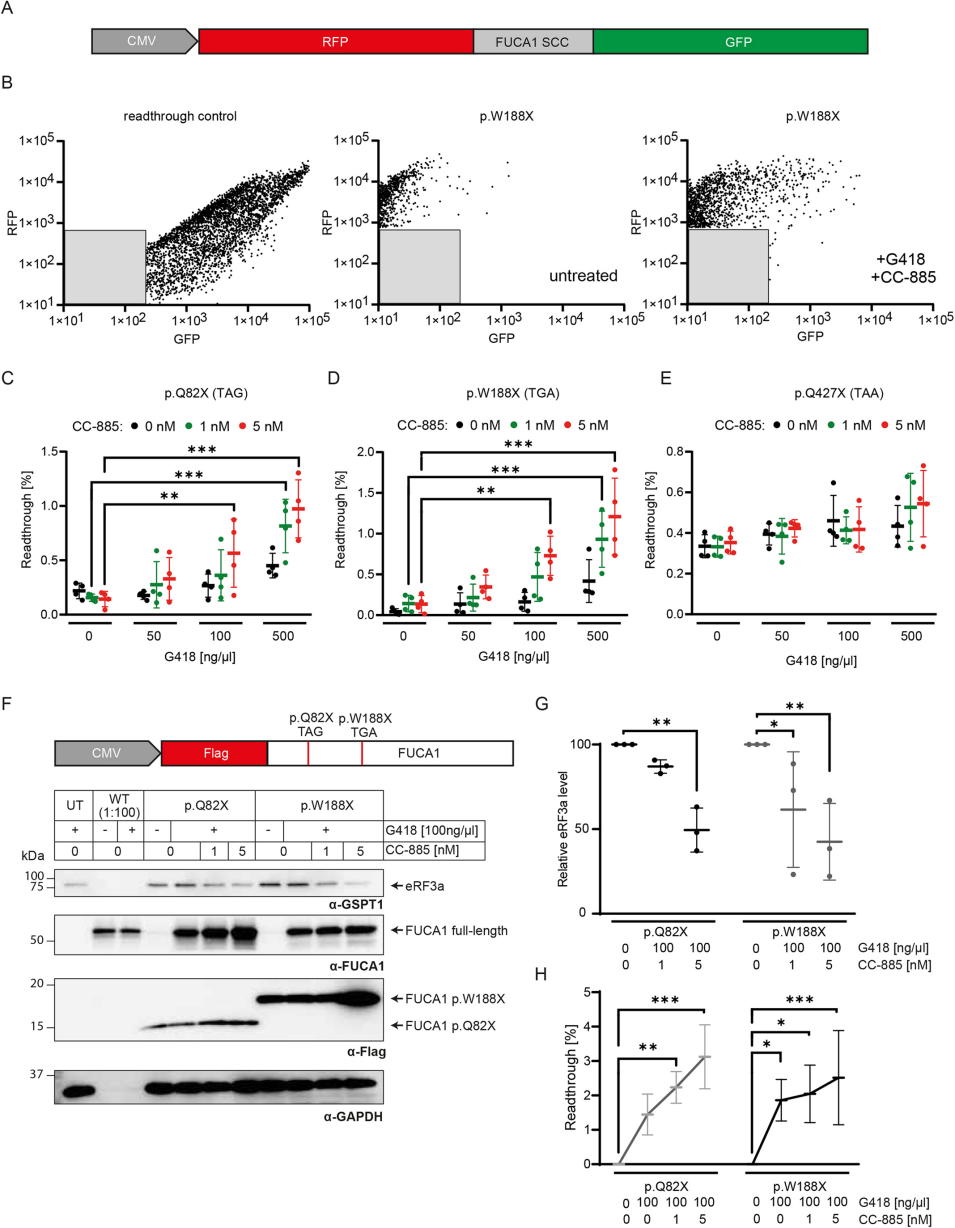
**Release factor degrader CC-885 boosts translational readthrough in *FUCA1* PTC mutations.** (A) Schematic dual reporter construct consisting of regions encoding TagRFP, the stop codon context (SCC) of FUCA1 p.Q82X, p.W188X or p.Q427X, and eGFP. (B) Gated cells displaying the fluorescence intensity of RFP and GFP for the 100% readthrough control (RFP-GFP fusion protein without a subdividing stop codon, left) or the p.W188X mutation under uninduced (middle) or G418 and CC-885-induced conditions (right). Gray boxes indicate untransfected HT1080 cells that were separated from further analysis. WT, wild type. (C-E) Translational readthrough quantification of the FUCA1 p.Q82X (C), p.W188X (D) or p.Q427X (E) SCC using various concentrations of G418 or CC-885 alone or in combination. *N*=4 biological replicates. Two-way ANOVA, with post hoc *P*-values from Bonferroni's test compared to cells with no G418 treatment. ***P<0.001; **P<0.01. Mid bar indicates mean; error bars indicate s.d. (F) Western blot was generated with lysates of transiently transfected cells, which were left untreated (−) or treated (+) with G418 and CC-885, and was analyzed by using anti-GSPT1 (detecting eRF3a), anti-Flag-tag and anti-FUCA1 antibodies. Anti-GAPDH antibody was used as a loading control. For WT, 0.25 µg total protein was loaded for SDS-PAGE; for all other samples, 25 µg of total protein was used per lane. Representative western blot from one of three biological replicates. (G,H) Densitometric quantification of visualized bands for eRF3a/GAPDH levels relative to the corresponding untreated sample (G) and full-length FUCA1 of p.Q82X or p.W188X relative to WT FUCA1 (1:100 dilution) to estimate readthrough efficacy (H). Two-way ANOVA, with post hoc *P*-values from Bonferroni's test compared to untreated cells. ***P<0.001; **P<0.01; *P<0.05. Mid bar indicates mean; error bars indicate s.d.

The efficacy of TR induction largely depends on the SSC including the stop codon. Using a fluorophore-based dual reporter assay (Lebeda et al., 2024) containing the 23-nucleotide *FUCA1* SCC (Fig. 2A), the effect of TR induction can be measured by flow cytometry (Fig. 2B). TR can be quantified by analysis of the GFP to RFP signal ratio, normalized to a 100% TR control (Fig. 2C-E). Here, we analyzed TR induction using G418 and simultaneous eRF3a degradation by CC-885 at various concentrations.

Without treatment, the TR was expectedly low at ∼0.2% for FUCA1 p.Q82X (Fig. 2C), ∼0.02% for p.W188X (Fig. 2D) and ∼0.34% for p.Q427X (Fig. 2E). Upon G418 treatment alone, no significant TR induction was observed at 50 ng/µl and 100 ng/µl, while only a slight effect could be observed at 500 ng/µl, increasing TR efficacy to ∼0.5% for FUCA1 p.Q82X and p.W188X.

The recovery of full-length FUCA1 depends on the concentration of G418, but high aminoglycoside concentrations are also associated with toxicity (Mingeot-Leclercq and Tulkens, 1999; Guthrie, 2008). To decrease toxicity and, at the same time, boost TR induction, we combined G418 treatment with the small molecule CC-885. CC-885 binds to the E3 ubiquitin-ligase complex consisting of cereblon (CRBN), damaged DNA binding protein 1 (DDB1) and cullin-4A (CUL4A), resulting in recognition, ubiquitination and degradation of eRF3a (Matyskiela et al., 2016). Here, the combined treatment with G418 and CC-885 led to dose-dependent TR induction. G418 in combination with 1 nM CC-885 increased the induction to ∼1% for the p.Q82X (Fig. 2C), p.W188X (Fig. 2D) and p.Q427X (Fig. 2E) SCCs. Increasing the CC-885 concentration to 5 nM further increased the inducibility to levels above 1% for FUCA1 p.Q82X and p.W188X. Affirming the lack of full-length recovery of FUCA1 p.Q427X, TR was also absent in the dual reporter with the p.Q427X SCC at any G418 concentration (Fig. 2E). Moreover, we could not observe significant recovery of FUCA1 activity upon high G418 treatment for this PTC (Fig. S1), so we excluded this mutation from further analyses. Cells treated with CC-885 alone showed no significant TR induction compared to non-treated cells.

Western blot analysis revealed efficient concentration-dependent degradation of eRF3a up to 50% using 5 nM CC-885 (Fig. 2F,G; Fig. S5). For the constructs p.Q82X and p.W188X, G418 alone recovered low levels of full-length FUCA1 between 1.5% and 2%. Upon dual treatment with 100 ng/µl G418 and 1 nM or 5 nM CC-885, the full-length recovery of FUCA1 was increased to up to 3%, depending on the concentration. The synergistic readthrough-stimulating effect of simultaneous eRF3a degradation was lower for the p.W188X SCC and more effective at the p.Q82X SCC (Fig. 2F,H; Fig. S5).

### Induction of translational readthrough restores PTC-containing FUCA1 signal and activity in HT1080 cells

**Fig. 3.**
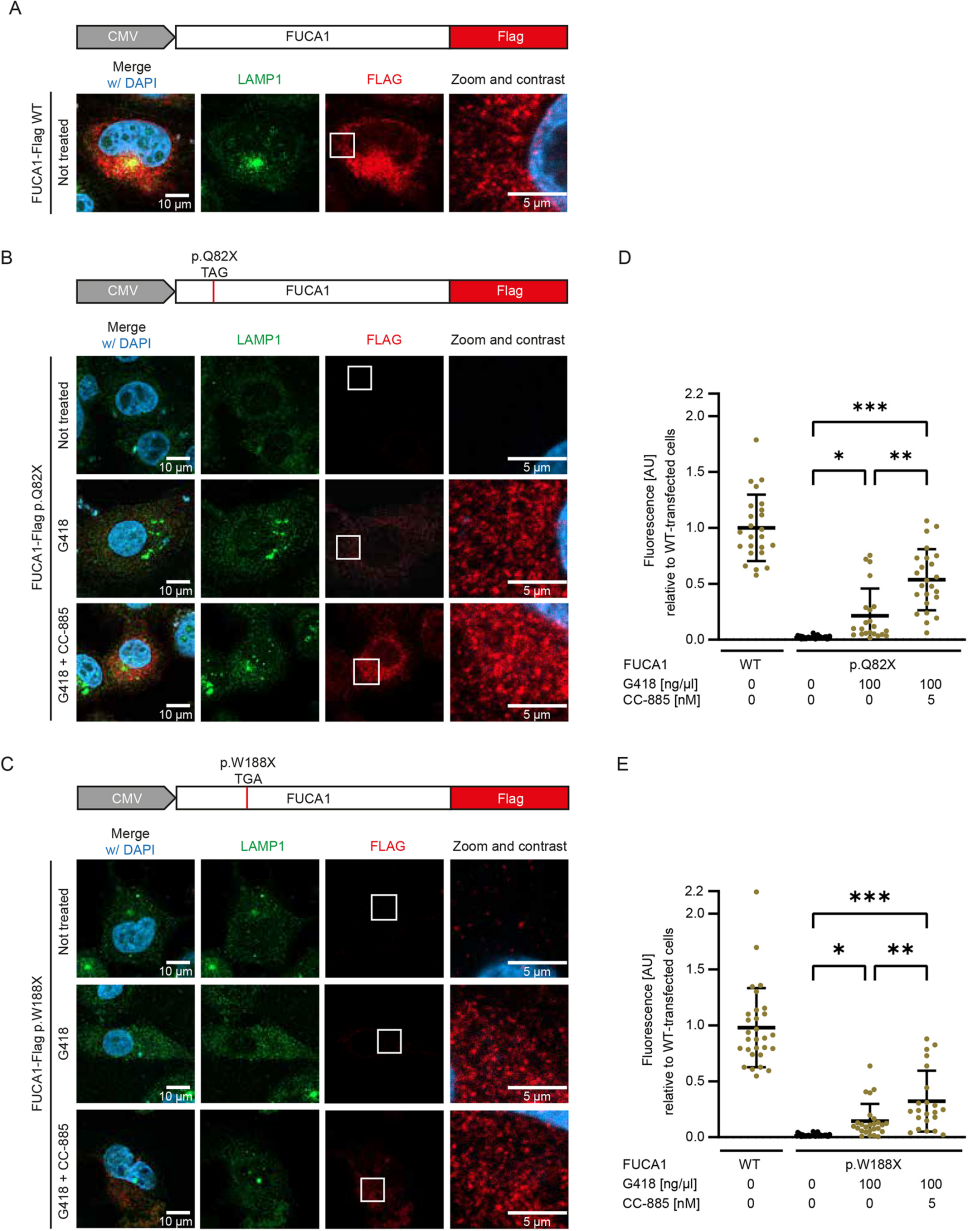
**Immunofluorescence of G418- and CC-885-treated and non-treated HT1080 cells.** Transient expression of FUCA1-Flag was detected using anti-Flag M2 antibody and Alexa Fluor 647 secondary antibody; endogenously expressed LAMP1 was detected using anti-LAMP1 antibody and Alexa Fluor 488 secondary antibody. (A) WT FUCA1-Flag expression in transfected HT1080 cells. (B,C) Transfection with FUCA1-Flag p.Q82X (B) or p.W188X (C) under non-treated conditions, or treatment with 100 ng/µl G418 alone or in combination with 5 nM CC-885. (D,E) Quantitative analysis of intracellular Flag signal normalized to the cell volume and relative to WT for p.Q82X (D) or p.W188X (E). AU, arbitrary units. *N*≥20. Two-way ANOVA, with post hoc *P*-values from Bonferroni's test compared to untreated cells. ***P<0.001; **P<0.01; *P<0.05. Mid bar indicates mean; error bars indicate s.d. Contrast, ‘Auto Brightness/Contrast’ algorithm in ImageJ.

So far, we had used dual reporter assays and western blotting to show readthrough induction with protein re-expression and recovery. The amino acid encoded by the stop codon upon readthrough is determined by a near-cognate tRNA (Hofhuis et al., 2016). As this amino acid may differ from the original amino acid, the protein may be dysfunctional, although it is full length. To analyze FUCA1 localization, we transfected HT1080 cells with plasmids encoding C-terminally Flag-tagged full-length FUCA1 containing either the WT sequence or the variants p.Q82X or p.W188X (Fig. 3). Cell viability assays showed a concentration-dependent cytotoxic effect of G418, whereas CC-885 exhibited no detectable toxicity within the tested range (Fig. S2). Based on these results, we selected 100 ng/µl G418 and 5 nM CC-885 as treatment conditions to maximize TR induction while minimizing cytotoxicity. Immunostaining of the C-terminal Flag-tag revealed the TR efficacy at the corresponding PTC and prevented the detection of truncated proteins. The localization pattern of the recovered full-length FUCA1 protein was compared to WT FUCA1-Flag and the lysosomal marker LAMP1 immunostaining. WT FUCA1-Flag associated with LAMP1 in proximate vesicular structures in HT1080 cells (Fig. 3A). Untreated FUCA1-Flag p.Q82X and p.W188X showed barely detectable fluorescence levels of ∼2.1% (p.Q82X) and ∼1.8% (p.W188X) of WT signal due to the low basal TR efficacy highlighted in the dual reporter assay and western blot analyses (Fig. 3B-E). Also, treatment with CC-885 alone did not lead to an increased detectable signal in any cell, indicating no inducible effect of eRF3a degradation alone (Fig. S3). However, treatment with G418 led to low recovery of intracellular FUCA1-Flag in both constructs (Fig. 3B,C). Signal intensity was ∼20%, compared to WT, for p.Q82X (Fig. 3D) and ∼10% for p.W188X (Fig. 3E). Furthermore, dual treatment with G418 and CC-885 increased the signal intensity to 50% and 40%, respectively, confirming the effect of eRF3a degradation in addition to readthrough induction. Of note, recovered FUCA1-Flag exhibited the same vesicular localization in proximity to LAMP1 structures as WT FUCA1-Flag, indicating correct lysosomal targeting. To quantify this spatial relationship, we analyzed proximity using object segmentation and measured center-to-center and edge-to-edge distances (Fig. S4). We consistently found spatial association between FUCA1-Flag and LAMP1 across all conditions. In all variants and treatments, ∼60% of FUCA1 signals were located within 0.2 µm of LAMP1-positive structures, and the mean center-to-center distance remained stable, closely resembling the wild-type distance of ∼0.35 µm. These findings confirm correct subcellular targeting of the rescued protein and reflect the expected lysosomal localization pattern, in which FUCA1 resides in the lumen and LAMP1 in the membrane.

**Fig. 4.**
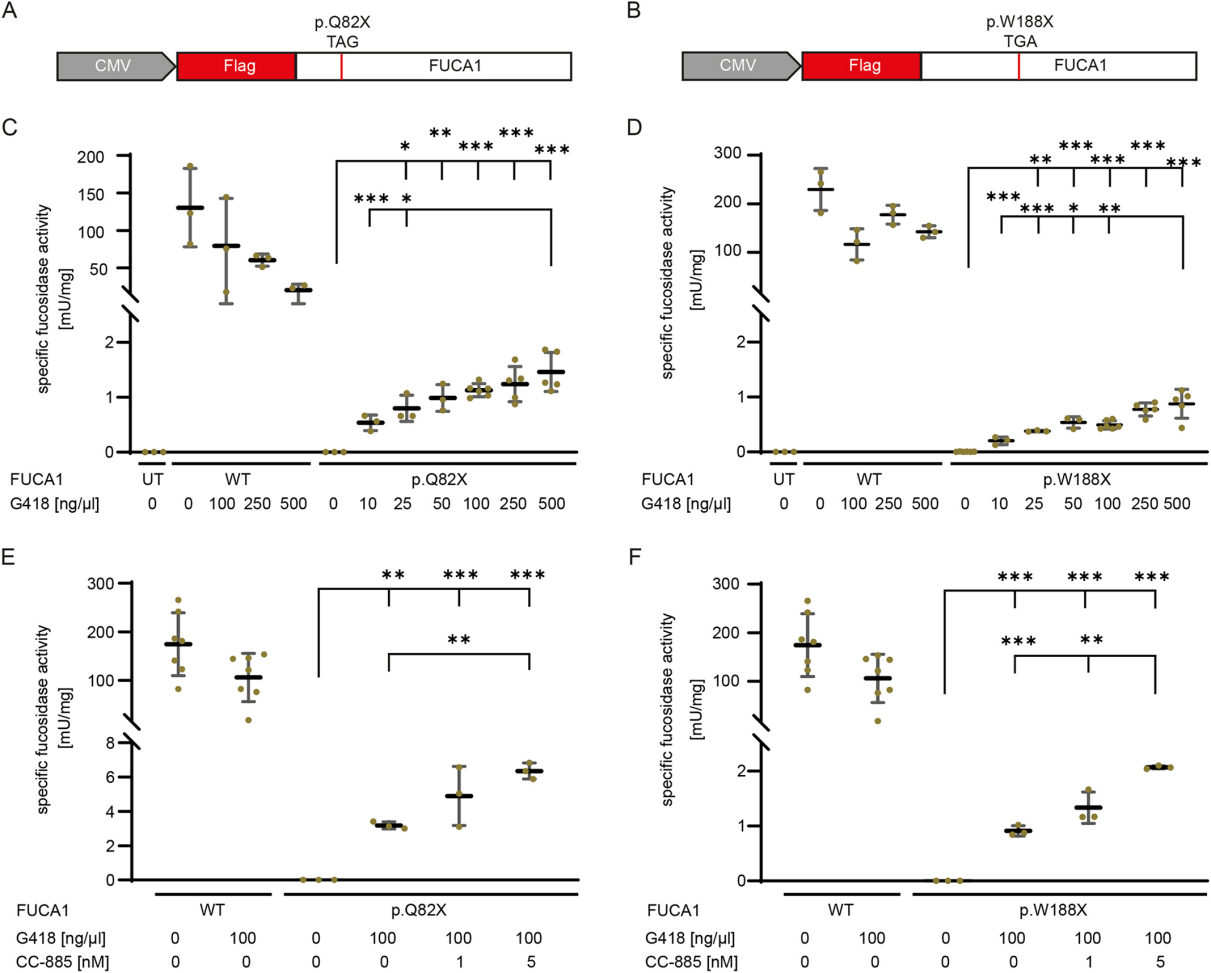
**Recovery of FUCA1 activity upon translational readthrough induction and release factor 3 degradation.** (A,B) HT1080 cells were transfected with plasmids encoding N-terminally Flag-tagged full-length FUCA1 bearing either no mutation, or the p.Q82X (A) or p.W188X (B) PTC. (C-F) FUCA1 activity assay in HT1080 cells, either untransfected or transfected with FUCA1-WT or a FUCA1 variant containing a PTC. Cells were treated with different G418 concentrations (C,D) or with 100 ng/µl G418 in combination with various CC-885 concentrations (E,F). *N*≥3 individual experiments. One-way ANOVA, with post hoc *P*-values from Bonferroni's test. ***P<0.001; **P<0.01; *P<0.05.

Because the recovery of FUCA1 localization alone does not guarantee the recovery of enzyme function, we measured FUCA1 activity in lysates of untreated and treated transfected cells. In untreated WT lysates, FUCA1 activity was between ∼100 and 300 mU/mg (Fig. 4C-F). G418 treatment compromised the FUCA1 activity in FUCA1-WT transfected cells in all measurements, probably due to its degrading effect on full-length FUCA1 that was shown by western blotting (Fig. 1B). FUCA1 activity was undetectable in the untreated p.Q82X (Fig. 4A,C,E) and p.W188X (Fig. 4B,D,F) variants. Treatment of p.Q82X and p.W188X with G418 alone recovered the FUCA1 activity to up to 1.46 mU/mg (Fig. 4C) and 0.88 mU/mg (Fig. 4D), respectively. We next investigated a potential increase in the FUCA1 activity by simultaneous G418 treatment and degradation of eRF3a by CC-885. The FUCA1 activity doubled with the addition of 5 nM CC-885 in comparison to that with only 100 ng/µl G418, resulting in 3.65% and 1.19% of the WT (not treated) activity in FUCA1 p.Q82X and p.W188X lysates (Fig. 4E,F), respectively. Combinatorial treatment with 1 nM CC-885 and 100 ng/µl G418 had only a minor effect on FUCA1 activity, resulting in slightly increased levels compared to those following treatment with G418 alone.

## DISCUSSION

In this study, we examined the use of TR induction at pathogenic PTCs in the *FUCA1* gene as a basis for the development of an individualized fucosidosis therapy. We created Flag-tagged reporter constructs containing WT or patient-like full-length *FUCA1* cDNA. Using the aminoglycoside G418, we induced TR and recovered full-length FUCA1. The strong TR response at p.Q82X (TAG) and p.W188X (TGA), in contrast to the weaker effect at p.Q427X (TAA), highlights the pivotal role of the SCC in modulating TR efficiency as previously reported (Lebeda et al., 2024; Schueren et al., 2014; Loughran et al., 2023; Schilff et al., 2021). The UGA stop codon is the most leaky stop codon exhibiting inducible TR levels of more than 1% and is therefore considered a favored stop codon for TR therapies, whereas UAA stop codons are known for their weak TR inducibility (Schueren et al., 2014; Hofhuis et al., 2016). Here, however, induction and full-length recovery did not significantly differ between p.W188X (TAG) and p.Q82X (TGA), although UGA stop codons generally exhibit a more efficient TR inducibility than TAG stop codons. This might be due to a weak SCC in the p.W188X context (Schueren et al., 2014). However, attributing specific effects to individual nucleotides within the SCC is difficult, as the underlying mechanisms by which the SCC modulates translational readthrough remain poorly understood. Recent findings further suggest that single nucleotides at defined positions do not exert uniform effects on TR. Instead, SCC nucleotides interact in a nonlinear fashion to determine TR efficiencies (Smoljanow et al., 2025).

As full-length recovery at all nonsense-mutated *FUCA1* variants is generally low when G418 concentrations are moderate, we investigated the simultaneous degradation of eRF3a by CC-885 to increase the probability of near-cognate tRNA incorporation at PTCs. This approach was shown to synergistically enhance TR efficacy at PTCs (Baradaran-Heravi et al., 2021; Heldin et al., 2023). Our data extend these findings to FUCA1, supporting the broader applicability of this approach to another LSD. Induction levels doubled upon eRF3a degradation for p.Q82X and p.W188X. Again, TR at the UAA stop codon for p.Q427X could not be induced owing to its generally high termination efficiency, although release factor degradation was successful at low concentrations of CC-885. Accordingly, full-length recovery of PTC-containing *FUCA1* constructs could be further increased by the dual treatment strategy, and only minor differences between p.Q82X and p.W188X constructs were visible despite the variation in stop codons. We have shown full-length recovery by western blotting and immunofluorescence. Although FUCA1-FLAG and LAMP1 do not show strong colocalization, distance-based analysis confirmed a consistent spatial association between the signals. This likely reflects the biological organization of lysosomes, as LAMP1 is embedded in the lysosomal membrane, whereas FUCA1 resides within the lumen. Lysosomes in HT1080 cells are typically <700 nm in diameter (Yordanov et al., 2019), which explains the observed average center-to-center distance of ∼350 nm between FUCA1 and LAMP1 signals.

We further tested the functionality of recovered full-length FUCA1, because TR could stimulate the integration of an unfavorable amino acid (Premchandar et al., 2024). Past studies found that Trp, Cys and Arg are favored for peptide chain incorporation at UGA stop codons, whereas Gln, Tyr and Lys are incorporated at UAG and UAA stop codons (Hofhuis et al., 2016; Blanchet et al., 2014; Schueren and Thoms, 2016; Zhang et al., 2024). We found concentration-dependent recovery in FUCA1 activity that depends on the corresponding nonsense mutation. However, WT samples treated with high G418 concentration showed reduced FUCA1 activity, likely due to degrading effects seen by western blotting. Simultaneous degradation of eRF3a significantly increased FUCA1 activity up to 3.65%. Although this increase highlights a promising synergistic effect of the dual treatment strategy, the level of enzymatic restoration remains modest. Furthermore, sustained or repeated degradation of eRF3a may impair global translation due to reduced termination efficiency, potentially disrupting protein synthesis and leading to deleterious cellular effects. Although CC-885 has been reported to induce cytotoxic effects in the low nanomolar range in other cell types, our viability assay showed no toxicity in HT1080 cells at a concentration of 5 nM (Baradaran-Heravi et al., 2021). However, future studies should further investigate the effects of sequential or long-term eRF3a depletion in more detail to evaluate its clinical relevance. We can, however, exclude a gross effect on translational readthrough at normal stop codons, because no significant increase in TR efficacy and full-length recovery can be observed using only CC-885 (Baradaran-Heravi et al., 2021).

Noteworthily, we investigated the effects of *FUCA1* PTCs on cDNA levels, so we did not include potential effects of NMD on the *FUCA1* transcript. According to the knowledge base StopKB, the p.Q82X and p.W188X variants are predicted to be sensitive to NMD, and future studies will profit from intron-containing constructs or cellular disease models (Haas et al., 2024). NMD inhibitors such as wortmannin and caffeine could stabilize nonsense-mutated transcripts, generating higher levels of full-length protein when combined with TR (Yamashita et al., 2001; Usuki et al., 2004; Keeling et al., 2013). Of note, only the induction of TR has been shown to significantly stabilize the transcript abundance indicating NMD escape by initializing the removal of exon-exon junctions from nonsense-mutated mRNA by the ribosome (Frew et al., 2020; Crawford et al., 2020).

TR presents a promising therapeutic strategy for fucosidosis patients, as a very-low percentage of the residual activity of the affected lysosomal enzyme might be sufficient to mitigate the clinical phenotype (Leinekugel et al., 1992; Conzelmann and Sandhoff, 1983). Although direct comparisons are limited by differing disease mechanisms, other LSDs illustrate how even minimal residual enzyme activity can significantly impact clinical outcomes. For example, patient-derived cells of the severe form (Hurler disease) of the LSD mucopolysaccharidosis type I (α-L-iduronidase deficiency) show α-L-iduronidase activities of below 0.15% of normal enzyme activity, while patient cells of the attenuated clinical form (Scheie disease) display between 0.3% and 7% of the normal enzyme activity (Bunge et al., 1998), indicating that small differences at the low end of the activity scale can make a considerable difference to the patient. In fucosidosis, a similar enzyme activity threshold may prevent storage of non-degraded substrates and thus lead to a delay or complete absence of clinical symptoms. Although the genotype-phenotype correlation is still unclear in fucosidosis (Willems et al., 1991, 1999, 1988; Stepien et al., 2020; Beratis et al., 1977), it is suggested that patients with the severe and fast-progressing form typically exhibit no FUCA1 activity (Willems et al., 1988; Stepien et al., 2020). Slightly higher activities (∼3-4%) have been observed in those with milder phenotypes, although systematic thresholds remain undefined (Beratis et al., 1977). Therefore, even modest increases in FUCA1 activity – as seen in our study – are of clinical relevance, especially if combined with early diagnosis and supportive therapy.

In this context, a high-throughput screening for TR inducibility of all 11 FUCA1 stop codons could serve as a valuable tool to systematically evaluate the TR potential. Our previously established dual-reporter assay enables the parallel assessment of TR efficiency across up to 96 variants per run based on their stop codon context (Lebeda et al., 2024). Although this system does not allow conclusions regarding full-length protein recovery or function, it provides an efficient platform to prioritize candidates for more in-depth validation using full-length expression constructs and functional assays.

The severe neurological involvement is the biggest challenge in fucosidosis therapy. Clinical experience shows that enzyme replacement therapy (ERT) does not sufficiently address these symptoms (Beck, 2018). A combination of ERT and TR could therefore provide a more effective treatment as some TR inducers are more prone to pass the blood-brain barrier than ERT proteins (Beryozkin et al., 2022; Fiduccia et al., 2024; Gurzeler et al., 2023). However, G418 cannot be considered a viable option for therapy owing to its toxicity. Further, G418 treatment led to FUCA1 degradation products, impaired enzymatic functionality and cell death, pointing out its disadvantageous use in fucosidosis. Future studies should investigate whether less toxic TR-inducing agents – such as aminoglycoside derivatives like ELX-02, or traditional aminoglycosides like gentamicin or amikacin – can synergize with eRF3a degradation to further enhance translational readthrough efficiency (Kandasamy et al., 2012; Du et al., 2006; Yang et al., 2025).

In conclusion, nonsense mutations in the *FUCA1* gene can be bypassed by treatment with aminoglycosides to restore the full-length version of the FUCA1 enzyme's activity. Future studies combining eRF3a degradation and less toxic and more efficient TR inducers (Brasell et al., 2019; Goldmann et al., 2012; Nudelman et al., 2009) might boost the TR efficacy to higher levels for a more efficient rescue effect. In turn, therapies for other PTC diseases will benefit from this strategy.

## MATERIALS AND METHODS

### Cloning of *FUCA1* expression constructs

We used the following constructs for this study: pcDNA3.1Hygro^(+)^-Flag-*FUCA1* (WT); pcDNA3.1Hygro^(+)^-Flag-*FUCA1-*Q82X; pcDNA3.1Hygro^(+)^-Flag-*FUCA1-*W188X; pcDNA3.1Hygro^(+)^-Flag-*FUCA1*-Q427X; pcDNA3.1Hygro^(+)^-*FUCA1-*Flag (WT); pcDNA3.1Hygro^(+)^-*FUCA1-*Q82X-Flag; pcDNA3.1Hygro^(+)^-*FUCA1-*W188X-Flag; pcDNA3.1Hygro^(+)^-*FUCA1*-Q427X-Flag; PST3394: Dual-Reporter_FUCA1-Q82X; PST3395: Dual-Reporter _FUCA1-W188X; PST3396: Dual-Reporter_FUCA1-Q427X.

The coding sequence for human *FUCA1* (NM_000147.5) was obtained from total RNA by reverse transcription followed by add-on PCR using specific primer pairs. All sequences were subcloned into the mammalian expression vector pcDNA3.1Hygro^(+)^ (Thermo Fisher Scientific) by using NheI and XhoI restriction sites as the add-on forward primers including a NheI site and the add-on reverse primers including a XhoI site. We included a Flag tag sequence either N-terminal (positioned between the signal sequence and P33 of the *FUCA1* coding sequence) or C-terminal (DYKDDDDK encoded by GACTACAAAGACGATGACGACAAG). Patient nonsense mutations were introduced by site-directed mutagenesis PCR (QuikChange II Site-Directed Mutagenesis Kit Instruction Manual, Agilent Technologies) using the following primers: Q82X forward, 5′-GTTCTGGTGGCACTGGTAGGGCGAGGGGC-3′; Q82X reverse, 5′-GCCCCTCGCCCTACCAGTGCCACCAGAAC-3′; W188X forward, 5′-CAATGCTGGGAATTTAAGGAGATCTGAAGTGG-3′; W188X reverse, 5′-CCACTTCAGATCTCCTTAAATTCCCAGCATT-3′; Q427X forward, 5′-CTCACTCTTAGAGTGATTCCATCCACTCTATC-3′; and Q427X reverse, 5′-GATAGAGTGGATGGAATCACTCTAAGAGTGAG-3′.

Dual reporter constructs were generated in pcDNA3.1(+)GFP_MCS_RFP (PST1596) as published (Hofhuis et al., 2017). The following oligonucleotides were used: OST3336 FUCA1Q82X_SCC-for, GTCACCGGTGGCACTGGTAGGGCGAGGGGCT; OST3337 FUCA1Q82X_SCC-rev, CCGGAGCCCCTCGCCCTACCAGTGCCACCG; OST3342 FUCA1W188X_SCC-for, GTCACCGACTCTTAGAGTGATTCCATCCACT; OST3343 FUCA1W188X_SCC-rev, CCGGAGTGGATGGAATCACTCTAAGAGTCG; OST3356 FUCA1Q427X_SCC-for, GTCACCGGCTGGGAATTTAAGGAGATCTGAT; OST3357 FUCA1Q427X_SCC-rev, CCGGATCAGATCTCCTTAAATTCCCAGCCG.

### Cell culture

HT1080 cells (American Type Culture Collection, CCL-121) were maintained in Dulbecco's modified Eagle medium (DMEM; Gibco) supplemented with 1% (v/v) glutamine (from a 200 mM stock; Bio&Sell), 1% (v/v) penicillin/streptomycin (from a 100× stock; Bio&Sell) and 10% (v/v) fetal calf serum (FCS; PAN Biotech) at 37°C in an atmosphere of 5% CO₂ and 90% humidity.

For immunofluorescence, 1×10^5^ cells were seeded per well on 18 mm coverslips in 12-well plates. For the dual reporter assay, 3×10^4^ cells were used per well in 96-well plates. Transfections were carried out using polyethylenimine (Kyofora Bio, 24765) with 150 ng plasmid DNA per well for 96-well plates (dual reporter assay) and 1 µg/well in 12-well plates (immunofluorescence). The transfection reagent was removed 16 h after transfection. Cells were subsequently treated with G418 (Carl Roth) and/or CC-885 (MedChemExpress) for 24 h.

For FUCA1 activity assays and western blot analyses, expression constructs were transiently expressed in HT1080 cells by standard polyethyleneimine transfection. 300,000 cells were seeded in six-well plates and were transfected with 2 µg/well plasmid DNA. Approximately 4.5 h after transfection, the culture medium was exchanged, and treatment was started as indicated.

### Preparation of cleared cell lysates and immunoblotting

Cells were harvested 24 h after transfection, lysed in Tris-buffered saline (TBS) with 0.5% Triton X-100 (v/v) by sonication on ice (3×10 s, duty cycle 80%, output control 8; Branson Sonifier 450) and cleared lysates were obtained by centrifugation (15,000 ***g***, 15 min, 4°C). After protein determination by detergent compatible assay (Bio-Rad), lysates were subjected to SDS-PAGE and analyzed by immunoblotting on polyvinylidene difluoride membrane and with antibodies directed against FUCA1 (our laboratory, MAB-A180; 1:500; Bäumges et al., 2025), Flag-tag (Cell Signaling Technology, D6WSB; 1:1000), GSPT1 (Thermo Fisher Scientific, PA5-62621; 1:1000) and GAPDH (Santa Cruz Biotechnology, sc-25778, FL-335; 1:500). Antibodies were diluted in TBS with 0.1% (w/v) Tween 20 and 5% (w/v) skimmed milk powder.

### α-L-fucosidase activity assay

α-L-fucosidase activity was measured using 4-methylumbelliferyl-α-L-fucopyranoside (4-MUF; Carbosynth). Cleared cell lysates were incubated with 150 μl of 0.75 mM 4-MUF in 0.1 M sodium citrate pH 5.5 including 0.2% bovine serum albumin (BSA). Reactions were incubated at 37°C and were stopped by the addition of 150 μl of 1 M sodium carbonate (pH 10.4). The amount of liberated 4-methylumbelliferone (4-MU) was determined by fluorescence measurements (excitation, 360 nm; emission, 465 nm) using a Spark 10M microplate reader (Tecan) and calculated using a standard curve (0 to 7.5 nmol 4-MU). Lysates were diluted 1:10 or 1:100 to reach a linear range.

### Immunofluorescence and microscopy

Transfected cells grown on 18 mm coverslips in 12-well plates were washed with sterile PBS and fixed with 4% paraformaldehyde (Thermo Fisher Scientific) for 10 min at room temperature (RT). After fixation, the cells were washed three times with PBS and permeabilized using 0.5% Triton X-100 (v/v) dissolved in PBS for 5 min at RT. Blocking was performed with 5% BSA in PBS for 30 min at RT to prevent non-specific antibody binding. The primary antibodies anti-Flag M2 (Merck, F1804; 1:1000) and anti-LAMP1 (Abcam, ab24170; 1:100) were diluted in PBS with 1% BSA, respectively. The coverslips were incubated overnight at 4°C. Goat anti-mouse and goat anti-rabbit secondary antibodies conjugated with Alexa Fluor 488 or Alexa Fluor 647 (Invitrogen, A32728, A11008, A32723, A21244) were diluted 1:1000 in PBS with 1% BSA, and with DAPI (0.1 μg/ml in PBS) for 1 h at RT. Coverslips were mounted with Fluorescence Mounting Medium (Dako, S3023) and left to dry overnight at RT. Images were recorded using a Zeiss LSM900 confocal fluorescence microscope with a 63× oil immersion objective. The following imaging parameters were used: Alexa Fluor 488, excitation at 493 nm, laser power at 1.5%, emission collected at 517 nm, detector gain 550 V; DAPI, excitation at 353 nm, laser power at 1.0%, emission collected at 465 nm, detector gain 500 V; Alexa Fluor 647, excitation at 653 nm, laser power at 1.5%, emission collected at 668 nm, detector gain 550 V. A 1 Airy Unit pinhole was used for all channels. Images were acquired in bidirectional scanning mode, with 4× line averaging and a digital gain of 1.0.

### Image analysis

Fiji-ImageJ software was used for image analysis and processing. For distance analysis of Flag to LAMP1 signals, fluorescence images were contrast-normalized (ImageJ ‘Auto Contrast’) prior to analysis. Background was subtracted using the rolling ball method (radius, 50 px). Signals were analyzed using the Phansalkar algorithm (radius, 15 px), followed by adaptive filtering (radius, 1 px; BioVoxxel plugin, accessed 4 August 2025) and segmentation via adaptive watershed (five erosion cycles; BioVoxxel). Distance measurements (center-to-center and edge-to-edge) were performed using the DiAna plugin in ImageJ (Gilles et al., 2017). Histograms and violin plots were generated in GraphPad Prism.

### Dual reporter assay and TR quantification

High-content dual reporter assay and TR quantification were carried out as previously described (Lebeda et al., 2024; Hofhuis et al., 2017). Cells were washed with 150 µl PBS and detached using 35 µl of 0.5% trypsin (Sigma-Aldrich) for 7 min. Cells were then resuspended in 165 µl Phenol Red-free DMEM supplemented with 1% penicillin/streptomycin and 10% FCS. Flow cytometry was performed in a 96-well plate format using the Guava EasyCyte 4th generation system (Luminex) using the 488 nm and 532 nm lasers. HT1080 cell gating was set based on forward scatter values between 23,000 and 75,000, and side scatter values between 12,500 and 67,500. Cells displaying RFP signal intensities above 900 or GFP signal intensities above 100 were included in TR calculations. TR was calculated as the ratio of GFP to RFP signal, normalized to the GFP/RFP ratio of a 100% TR control vector that expresses an RFP-GFP fusion protein without a separating stop codon (PST1596). For each 96-well plate, PST1596 and untransfected HT1080 cells were measured as controls. Gating and calculations were performed based on the corresponding FCS3.0 file generated after each measurement using RStudio (R Core Team).

### Cell viability assay

To assess cytotoxic effects of G418 and CC-885, we performed a colorimetric assay with the Orangu™ reagent (Cell Guidance Systems). Cells were seeded in a 96-well plate at a density of 5000 cells/well. Cells were incubated overnight at 37°C in an atmosphere of 5% CO₂ and 90% humidity to achieve cell adherence. Cells were treated with G418 (range, 0-500 ng/µl) and CC-885 (range, 0-5 nM) alone or in combination at a final volume of 90 µl. After 24 h, 10 µl Orangu cell counting solution was added, and cells were incubated under the same conditions. After 2 h, absorbance was measured at 450 nm. Cell viability was determined as the absorbance of each well normalized to that of the untreated controls.

### Statistical analysis

All experiments were performed in triplicates. When more than three replications were performed, it is stated in the figure panel description. Data were analyzed with GraphPad Prism software. Scatter plots indicate mean with individual values; error bars indicate s.d.

## Supplementary Material

10.1242/dmm.052495_sup1 Supplementary information
