# Supplementary material for: Recovery of α-L-fucosidase in fucosidosis nonsense variants by readthrough stimulation and release factor degradation
Source: Dis Model Mech. 2026 Jan 7;19(1):dmm052495. doi: 10.1242/dmm.052495 (PMC12817336; doi:10.1242/dmm.052495)
Supplement: Supplementary information [file dmm-19-052495-s1.pdf]

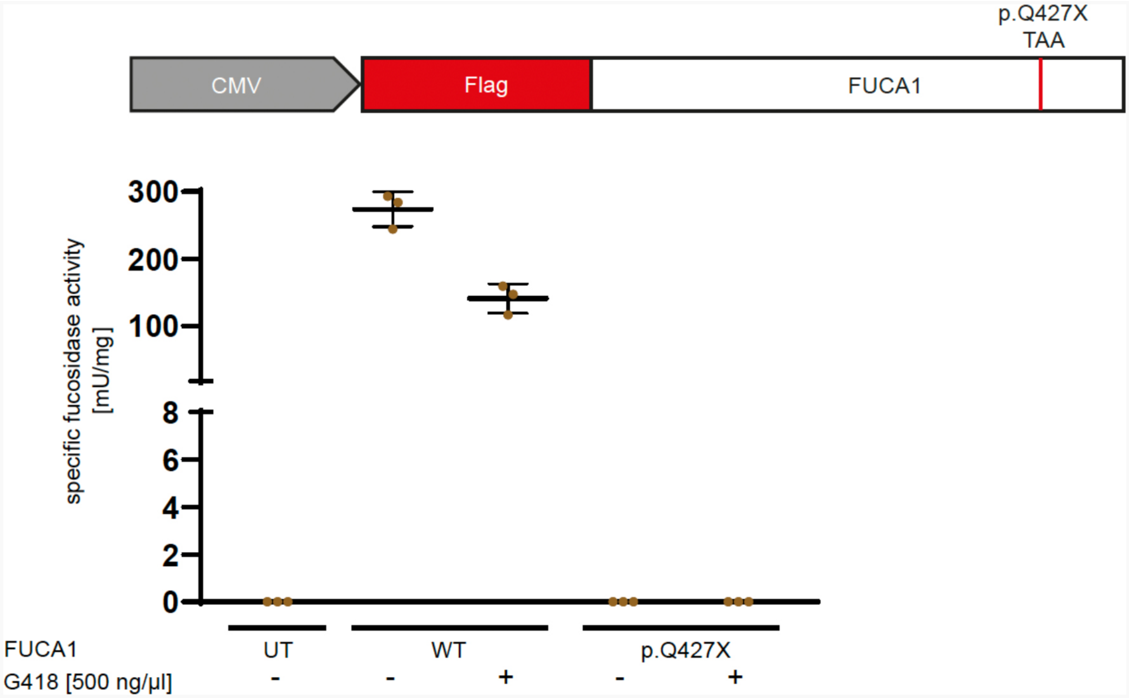

Fig. S1. α-L-fucosidase activity assay of HT1080 cells transfected with plasmids encoding N-terminally Flag-tagged full-length FUCA1 bearing either no mutation or the p.Q427X PTC. Untransfected (UT) and p.Q427X-transfected cells show no α-L-fucosidase activity. The activity in WT-transfected cells decrease upon treatment with a high G418 concentration and no increase of FUCA1 p.Q427X activity is measurable.

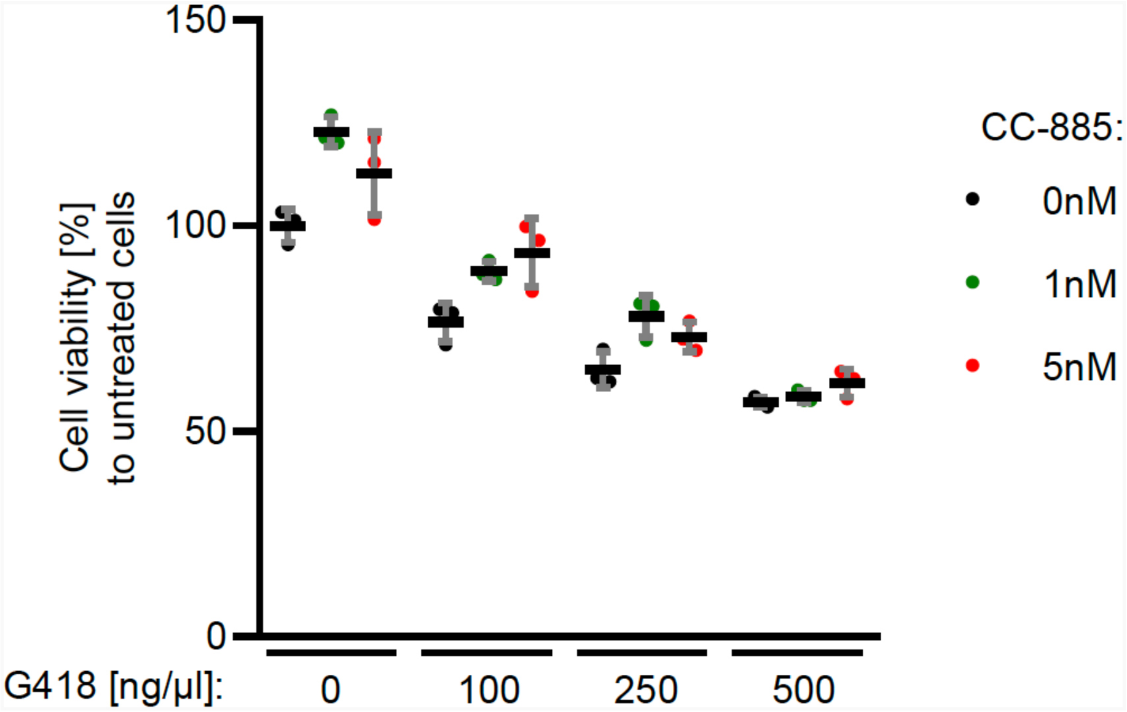

Fig. S2. Cell viability of HT1080 cells after 24 hours treatment of G418 and CC-885 alone or in combination. Viability was assessed using the Orangu™ cell counting assay. Orangu reagent was added to each well and incubated for 2 h, followed by absorbance measurement at 450 nm. Data represent mean ± SD of three biological replicates. Absorbance values were normalized to untreated controls.

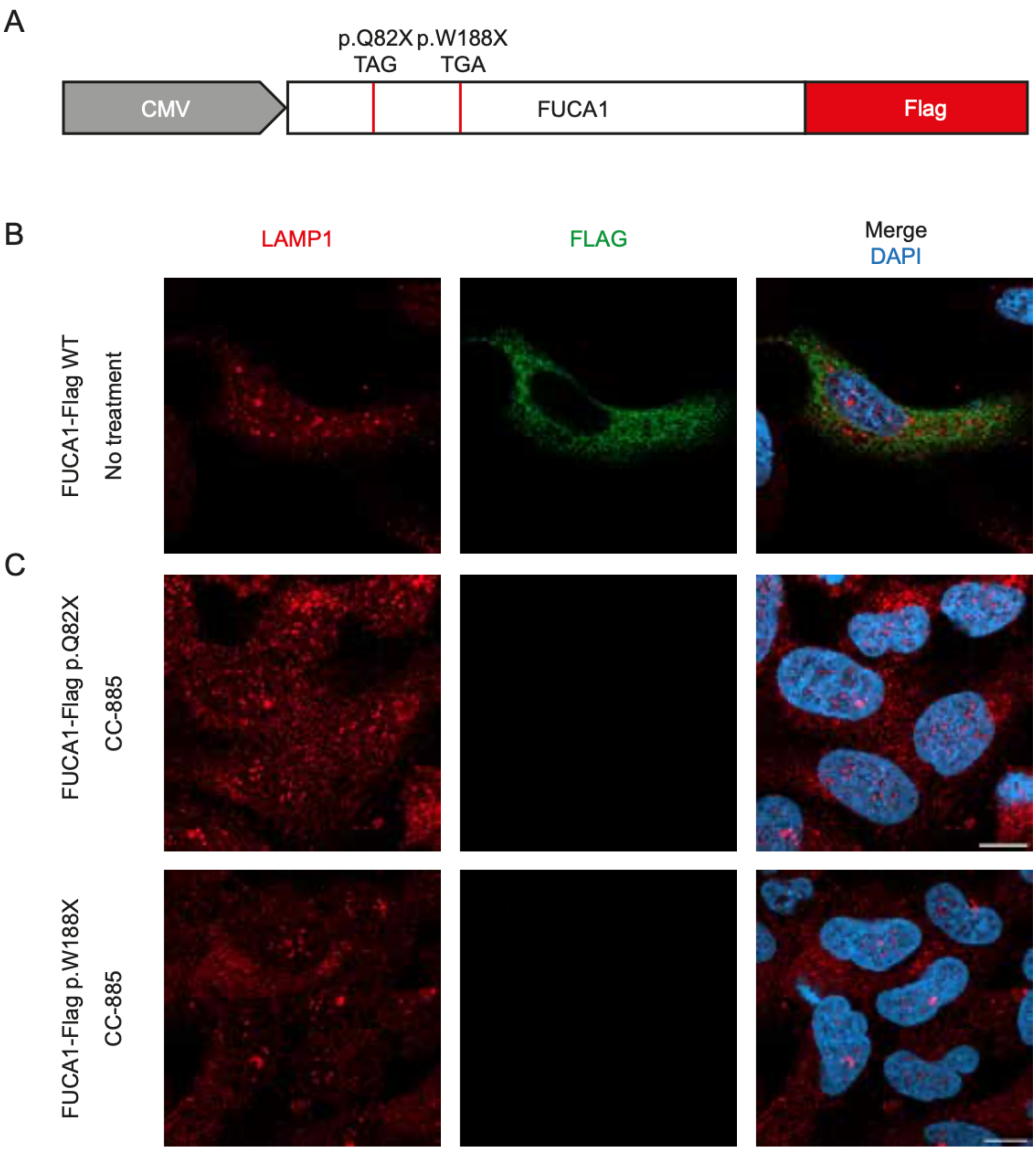

Fig. S3. Transient expression of FUCA1-FLAG was detected using anti-FLAG M2 antibody and Alexa Fluor 488 secondary antibody while endogenously expressed LAMP1 was detected using LAMP1 antibody and Alexa Fluor 647 secondary antibody. A: Cells were transfected with plasmids encoding C-terminally Flag-tagged full-length FUCA1 bearing no mutation, the p.Q82X or the p.W188X PTC. **B:** Wildtype FUCA1-FLAG expression in HT1080 cells. **C:** Transfection with either FUCA1-Flag p.Q82X nor p.W188X leads to no visible expression when treated with 5nM CC-885. Scale bar = 10 µm.

Disease Models & Mechanisms • Supplementary information

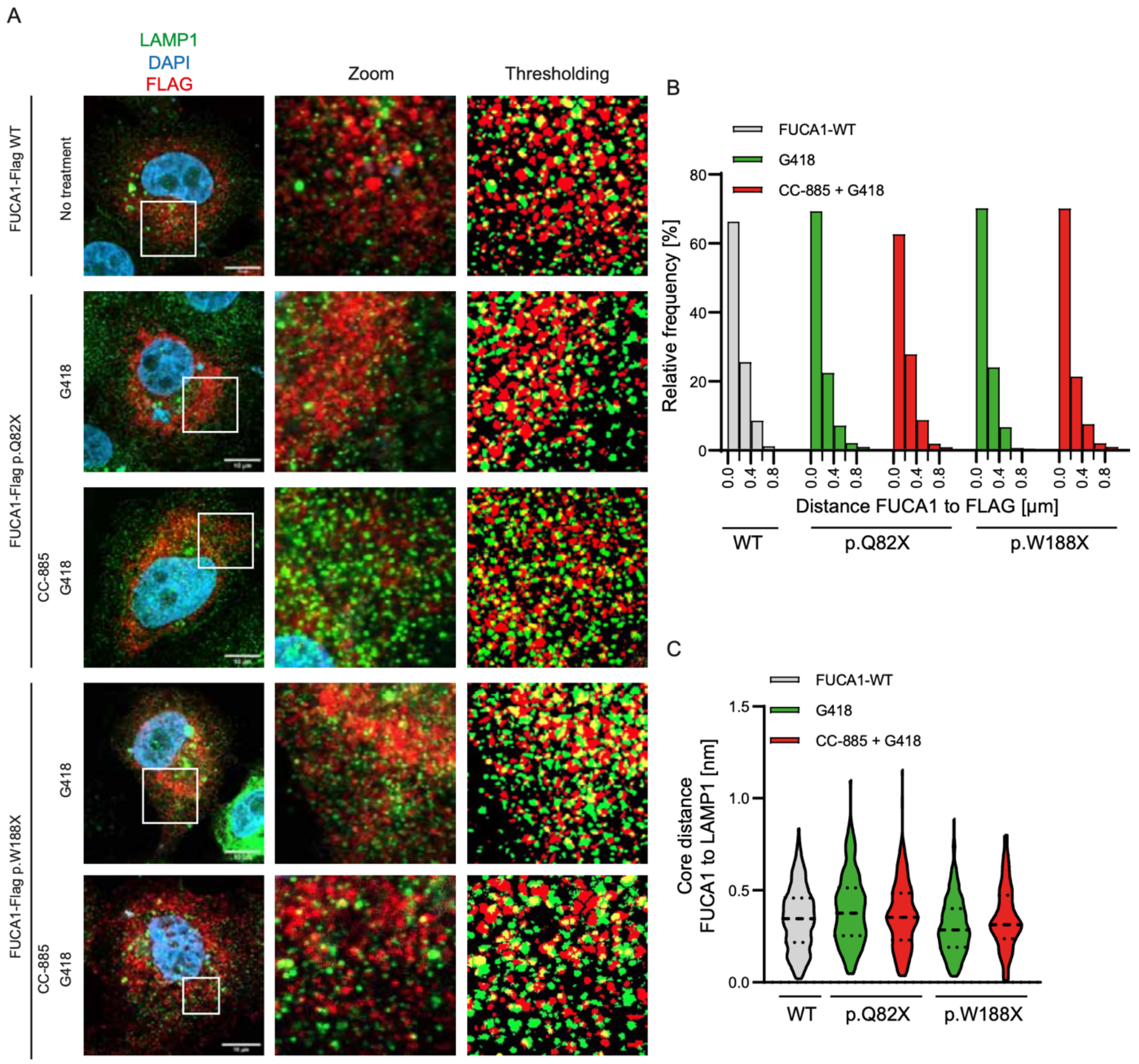

Fig. S4. Distance analysis between transiently expressed FUCA1-FLAG and endogenous LAMP1. A: Immunofluorescence images of HT1080 cells transiently expressing FUCA1-FLAG WT, p.Q82X, or p.W188X. Cells were untreated or treated with G418, CC-885, or both. Signal intensities were set to uniform level. Zoomed insets (white boxes) show regions used for quantitative analysis. Thresholded images illustrate segmented FLAG and LAMP1 signals. **B:** Edge-to-edge distances (bin width: 0.2 µm) between FLAG and LAMP1 signals. **C:** Violin plots of center-to-center distances between segmented FUCA1-FLAG and LAMP1 objects. Interrupted bars indicate mean, dotted lines indicate quartiles.

Disease Models & Mechanisms • Supplementary information

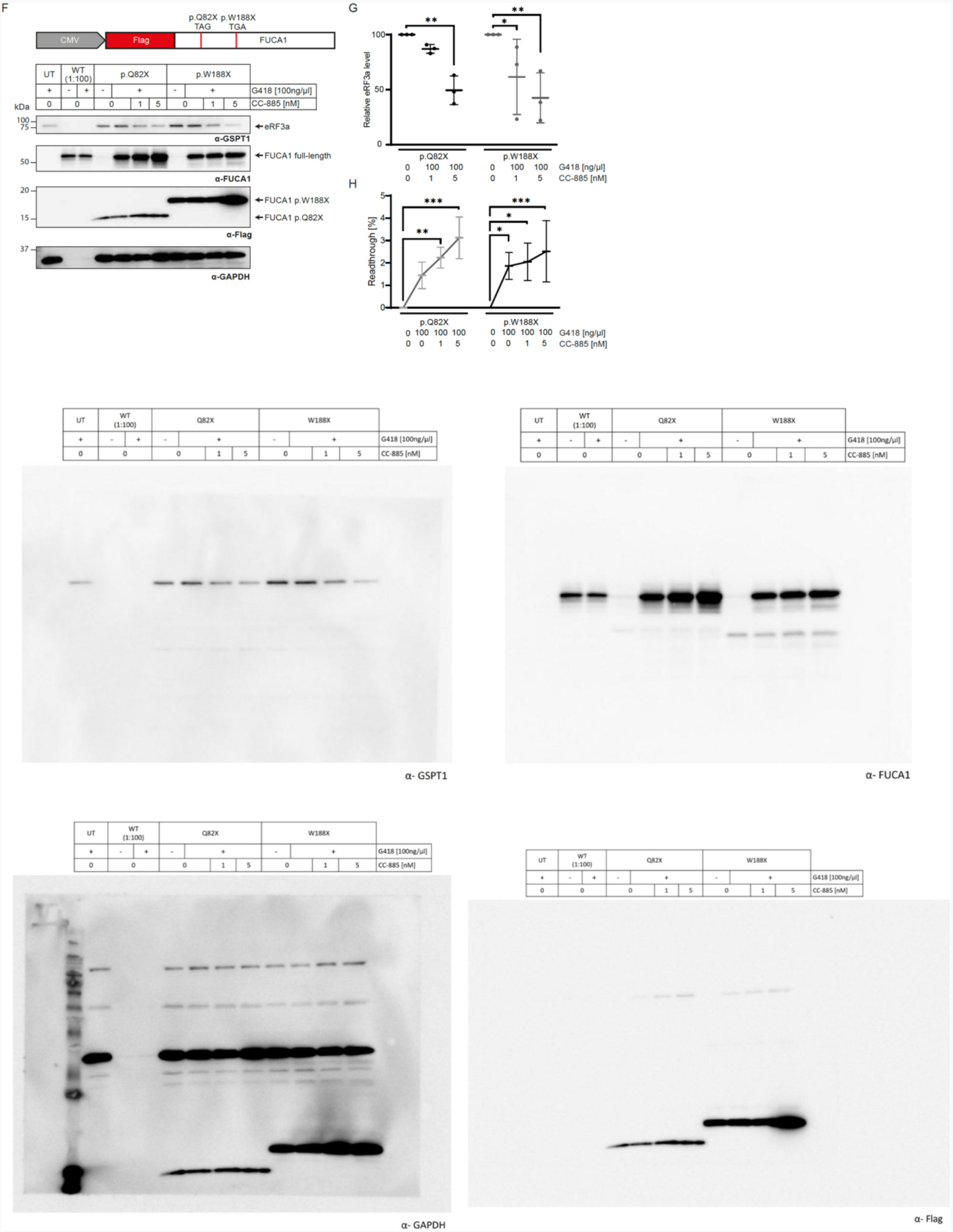
F
CMV
Flag
FUCA1
p.Q82X TAG
p.W188X TGA
UT
WT (1:100)
p.Q82X
p.W188X
G418 [100ng/µl]
CC-885 [nM]
kDa
eRF3a
α-GSPT1
FUCA1 full-length
α-FUCA1
FUCA1 p.W188X
FUCA1 p.Q82X
α-Flag
α-GAPDH
G
Relative eRF3a level
p.Q82X
p.W188X
G418 [ng/µl]
CC-885 [nM]
H
Readthrough [%]
p.Q82X
p.W188X
G418 [ng/µl]
CC-885 [nM]
α- GSPT1
α- FUCA1
α- GAPDH
α- Flag

G

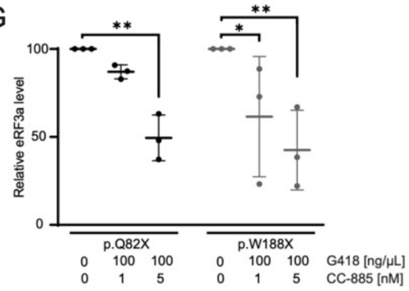

H

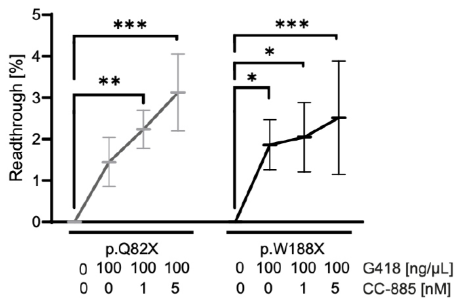

blots for quantification #2

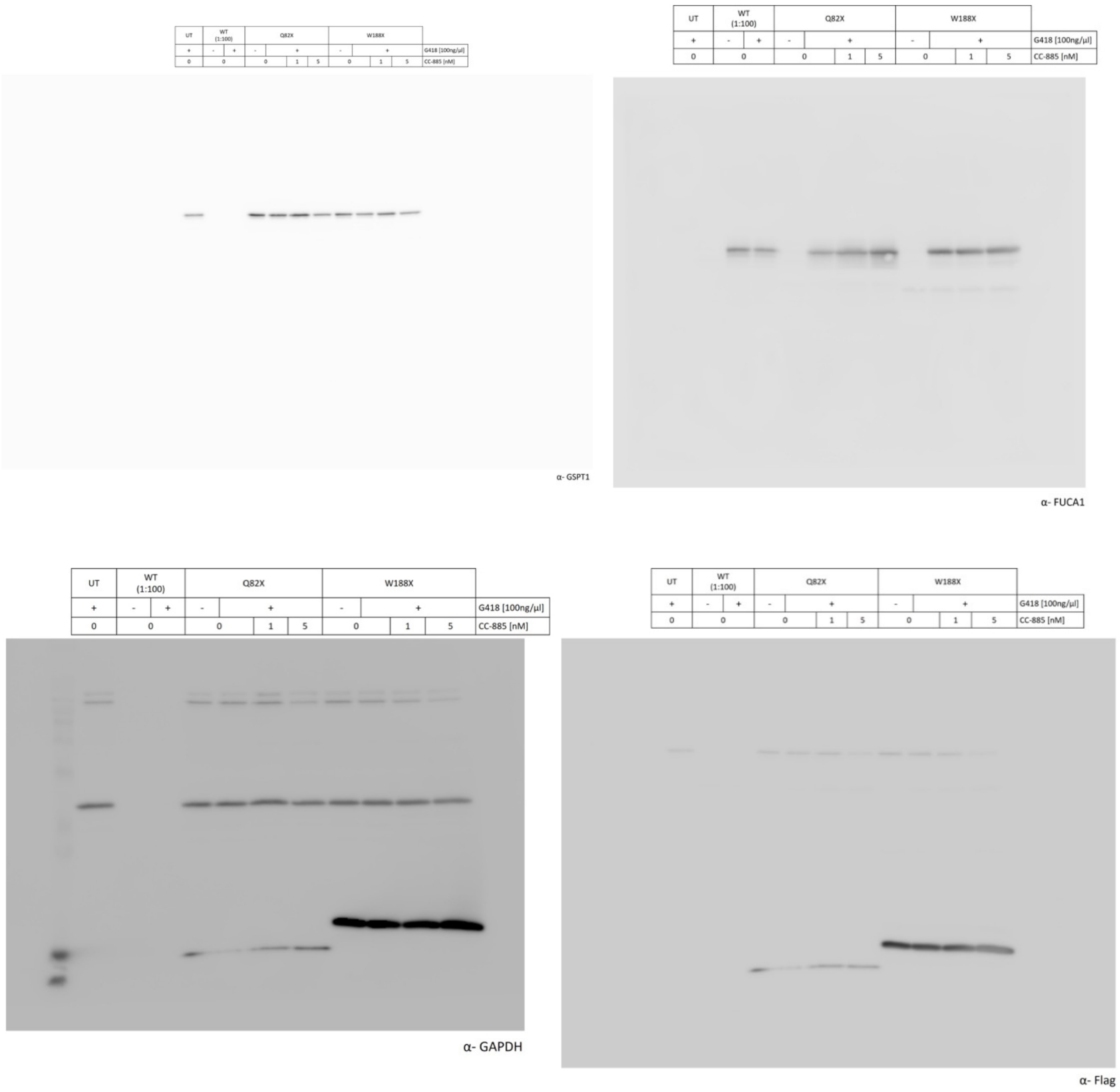

Disease Models & Mechanisms • Supplementary information

Figure 2
G & H:

blots for quantification #3

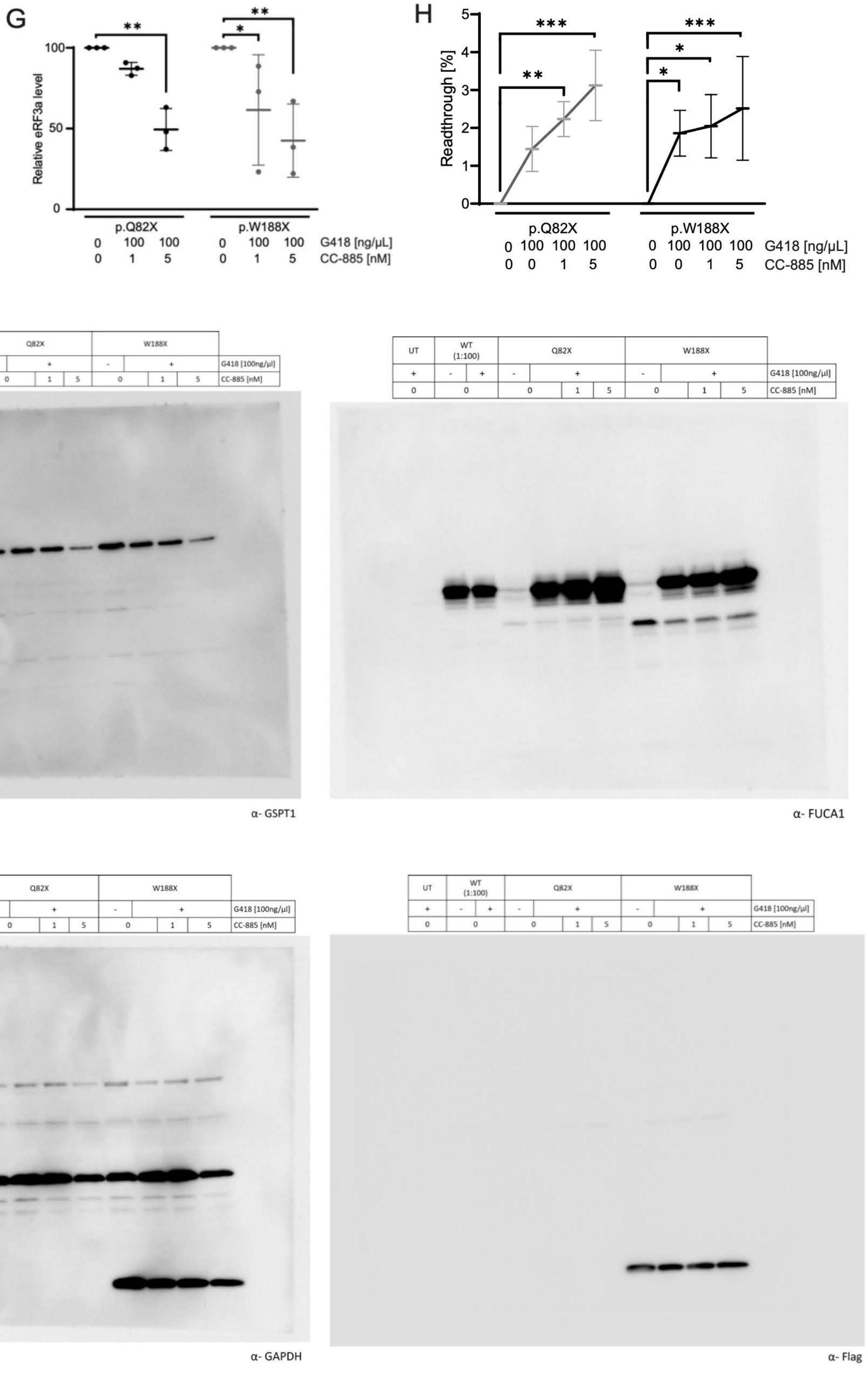

Fig. S5. Unedited Western Blots used for quantification in Figure 2 G & H.

Disease Models & Mechanisms • Supplementary information
